# Sorafenib Sensitizes Solid Tumors to Apo2L/TRAIL and Apo2L/TRAIL Receptor Agonist Antibodies by the Jak2-Stat3-Mcl1 Axis

**DOI:** 10.1371/journal.pone.0075414

**Published:** 2013-09-26

**Authors:** Junaid Abdulghani, Joshua E. Allen, David T. Dicker, Yingqiu Yvette Liu, David Goldenberg, Charles D. Smith, Robin Humphreys, Wafik S. El-Deiry

**Affiliations:** Laboratory of Translational Oncology and Experimental Cancer Therapeutics, Department of Medicine (Hematology/Oncology), Penn State Hershey Cancer Institute, Penn State College of Medicine, Hershey, Pennsylvania, United States of America; Department of Surgery, Penn State Hershey Cancer Institute, Penn State College of Medicine, Hershey, Pennsylvania, United States of America; Pharmaceutical and Biomedical Sciences Department, College of Pharmacy, Medical University of South Carolina, Charleston, South Carolina, United States of America; Oncology Research Department, Human Genome Sciences, Rockville, Maryland, United States of America; Bauer Research Foundation, United States of America

## Abstract

**Background:**

Approximately half of tumor cell lines are resistant to the tumor-selective apoptotic effects of tumor necrosis factor-related apoptosis-inducing ligand (Apo22L/TRAIL). Previously, we showed that combining Apo2L/TRAIL with sorafenib, a multikinase inhibitor, results in dramatic efficacy in Apo2L/TRAIL-resistant tumor xenografts via inhibition of Mcl-1. Soluble Apo2L/TRAIL is capable of binding to several surface receptors, including the pro-apoptotic death receptors, DR4 and DR5, and decoy receptors, DcR1 and DcR2. Monoclonal antibodies targeting either of these death receptors are being investigated as antitumor agents in clinical trials. We hypothesized that sorafenib and Apo2L/TRAIL or Apo2L/TRAIL death receptor agonist (TRA) antibodies against DR4 (mapatumumab) and DR5 (lexatumumab) will overcome resistance to Apo2L/TRAIL-mediated apoptosis and as increase antitumor efficacy in Apo2L/TRAIL-sensitive solid tumors.

**Methodology/Principal Findings:**

We found that Apo2L/TRAIL or TRA antibodies combined with sorafenib synergistically reduce cell growth and increase cell death across a panel of solid tumor cell lines in vitro. This panel included human breast, prostate, colon, liver and thyroid cancers. The cooperativity of these combinations was also observed *in* *vivo*, as measured by tumor volume and TUNEL staining as a measure of apoptosis. We found that sorafenib inhibits Jak/Stat3 signaling and downregulates their target genes, including cyclin D1, cyclin D2 and Mcl-1, in a dose-dependent manner.

**Conclusions/Significance:**

The combination of sorafenib with Apo2L/TRAIL or Apo2L/TRAIL receptor agonist antibodies sensitizes Apo2L/TRAIL-resistant cells and increases the sensitivity of Apo2L/TRAIL-sensitive cells. Our findings demonstrate the involvement of the Jak2-Stat3-Mcl1 axis in response to sorafenib treatment, which may play a key role in sorafenib-mediated sensitization to Apo2L/TRAIL.

## Introduction

Solid tumors cause several hundred thousand deaths each year in the United States [1,2]. Surgery, radiation and chemotherapy have been the mainstay of cancer treatment with removal of the cancer without damage to the rest of the body as the goal of treatment. Cancers tend to invade adjacent tissue or spread to distant sites by micrometastases, leading to morbidity and mortality. Ongoing efforts to improve chemotherapy involve rationally designed therapies that target tumor-selective cell death pathways that spare normal cells. Tumor necrosis factor-related apoptosis-inducing ligand (Apo2L/TRAIL) has been identified as one such target [3]. Apo2L/TRAIL can activate the extrinsic pathway of cell death by binding to the death receptors, DR4 and DR5; in addition, Apo2L/TRAIL can bind to the decoy receptors, DcR1 and DcR2 which lack intracellular death domains and therefore do not induce cell death [4]. After binding Apo2L/TRAIL, the death receptors form homotrimers to recruit Fas-associated protein with death domain (FADD). This recruits caspase-8 to form the death-inducing signaling complex (DISC), resulting in the activation of caspase-8. Activated caspases-8 can then cleave the effector caspase-3, which proceeds to cleave death substrates [5]. Phase I/II clinical trials have been completed with a fully-human anti-DR4 agonist antibody (mapatumumab) in non-small-cell lung carcinoma and Non-Hodgkin’s lymphoma and phase 1 clinical trials with anti- DR5 monoclonal antibody (lexatumumab) in advanced cancers [6,7,8]. Mapatumumab was found to be safe and well tolerated at concentrations of 10 mg/kg body weight. In contrast to the recombinant ligand that has a serum half-life of approximately 30 minutes, these antibodies have a significantly increased serum half-life of approximately 1-2 weeks.

Sorafenib is a multikinase inhibitor that acts on a number of kinases including Raf Kinases, MEK, ERK signaling as well as on vascular endothelial growth factor receptor 2 (VEGFR2), platelet derived growth factor receptor (PDGFR), FLT3, Ret and c-Kit [9]. Recently, sorafenib (Nexavar) was approved for the treatment of unresectable liver and advanced renal cancer [10,11]. We have previously shown that the sensitivity of cancer cells to Apo2L/TRAIL-mediated cell death is greatly increased when the anti-apoptotic Bcl-2 family member Mcl-1 is downregulated by sorafenib [12]. Mcl-1 is considered a critical gateway for Apo2L/TRAIL sensitization, and Mcl-1 might cause Apo2L/TRAIL resistance by acting as a buffer for Bak, Bim and PUMA [12,13]. Mcl-1 is over-expressed in a number of solid tumors, and therefore, it represents a considerable resistance barrier for Apo2L/TRAIL as an antitumor agent [14,15].

Stat3 is both a cytoplasmic signaling molecule and a nuclear transcription factor that is activated by the phosphorylation of a specific tyrosine residue in its carboxy-terminal by Jak kinases in response to cytokines, including IL-6, IFN, epidermal growth factor, and FGF [16,17]. In the nucleus, Stat3 regulates the expression of the proteins that regulate mitochondrial-mediated apoptosis, such as Bcl-2, Mcl-1 and cIAP2 [18]. In this study, we show that sorafenib sensitizes Apo2L/TRAIL-resistant cancer cells and enhances cell death in Apo2L/TRAIL-sensitive solid tumors in combination with mapatumumab, lexatumumab, or Apo2L/TRAIL. We found that sorafenib-mediated sensitization to Apo2L/TRAIL may involve the Jak2-Stat3-Mcl-1 axis in solid tumors.

## Results

### Sorafenib and lexatumumab monotherapies induce cell death in hepatocellular cancer cell lines *in vitro*

**Figure 1:**
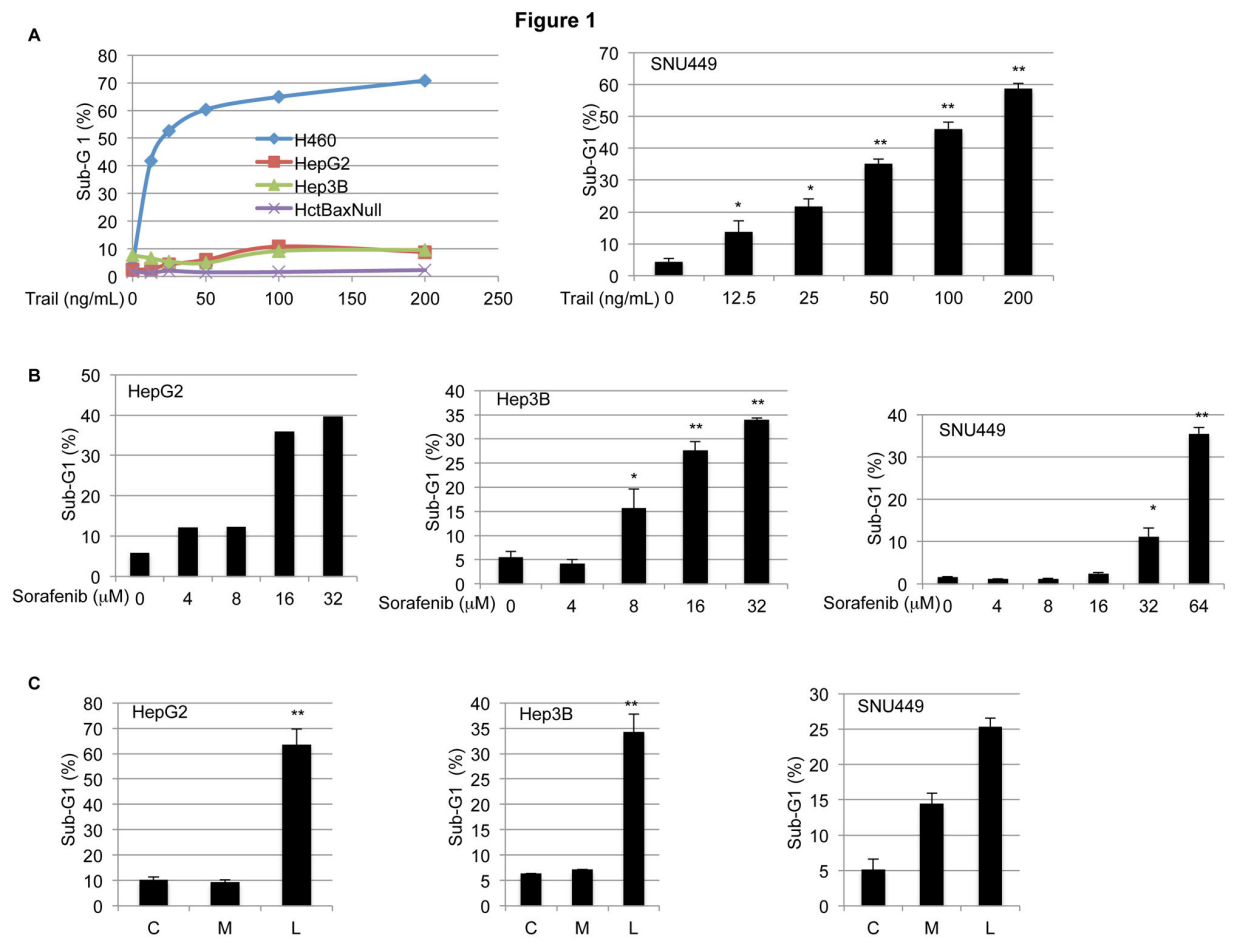
Sorafenib and lexatumumab as monotherapies induce cell death in hepatocellular carcinoma cell (HCC) lines *in vitro*. A. HepG2, Hep3B and SNU449 cells were treated with increasing concentration of Apo2L/TRAIL (12.5, 25, 50, 100, 200 ng/mL) for 24 h. Percentage of cells with sub-G1 content are shown. HepG2 and Hep3B cells are resistant to Apo2L/TRAIL whereas SNU449 is sensitive to Apo2L/TRAIL (n=3, mean+S.D., *p<0.05, **p<0.005). B. HCC cell lines were treated with increasing concentrations of sorafenib (4, 8, 16, 32 µM) for 24 h (HepG2, Hep3B) and 48 h (SNU449) and analyzed for sub-G1 content. All cell lines tested were sensitive to sorafenib. C. Cells were treated with Apo2L/TRAIL receptor agonist antibodies (TRA), mapatumumab (1000 ng/mL) or lexatumumab (1000 ng/mL) for 24 h and sub-G1 content was analyzed (C=Control, M=mapatumumab, L=lexatumumab)..

We have previously shown that sorafenib sensitizes Apo2L/TRAIL-resistant cancer cells by down-regulating the expression of Mcl-1 [12]. We therefore examined the sensitivity of liver cancer cells to Apo2L/TRAIL. HepG2 and Hep3B cell lines were resistant to Apo2L/TRAIL at concentrations up to 200 ng/mL, and the SNU449 cell line was sensitive to Apo2L/TRAIL in a concentration-dependent manner (Figure 1A). We used the Apo2L/TRAIL-sensitive lung carcinoma cell line H460 and the Apo2L/TRAIL-resistant colon carcinoma cell line HCT116 Bax^-/-^ as positive and negative controls, respectively [19,20]. However, all of the tested liver cancer cell lines were sensitive to sorafenib in a concentration-dependent manner, as determined by sub-G1 analysis (Figure 1B). We found that these cell lines were sensitive to lexatumumab as monotherapy but not to mapatumumab under these experimental conditions (Figure 1C).

### Sorafenib sensitizes human hepatocellular carcinoma cell lines to cell death induced by Apo2L/TRAIL or Apo2L/TRAIL receptor agonist antibodies

**Figure 2:**
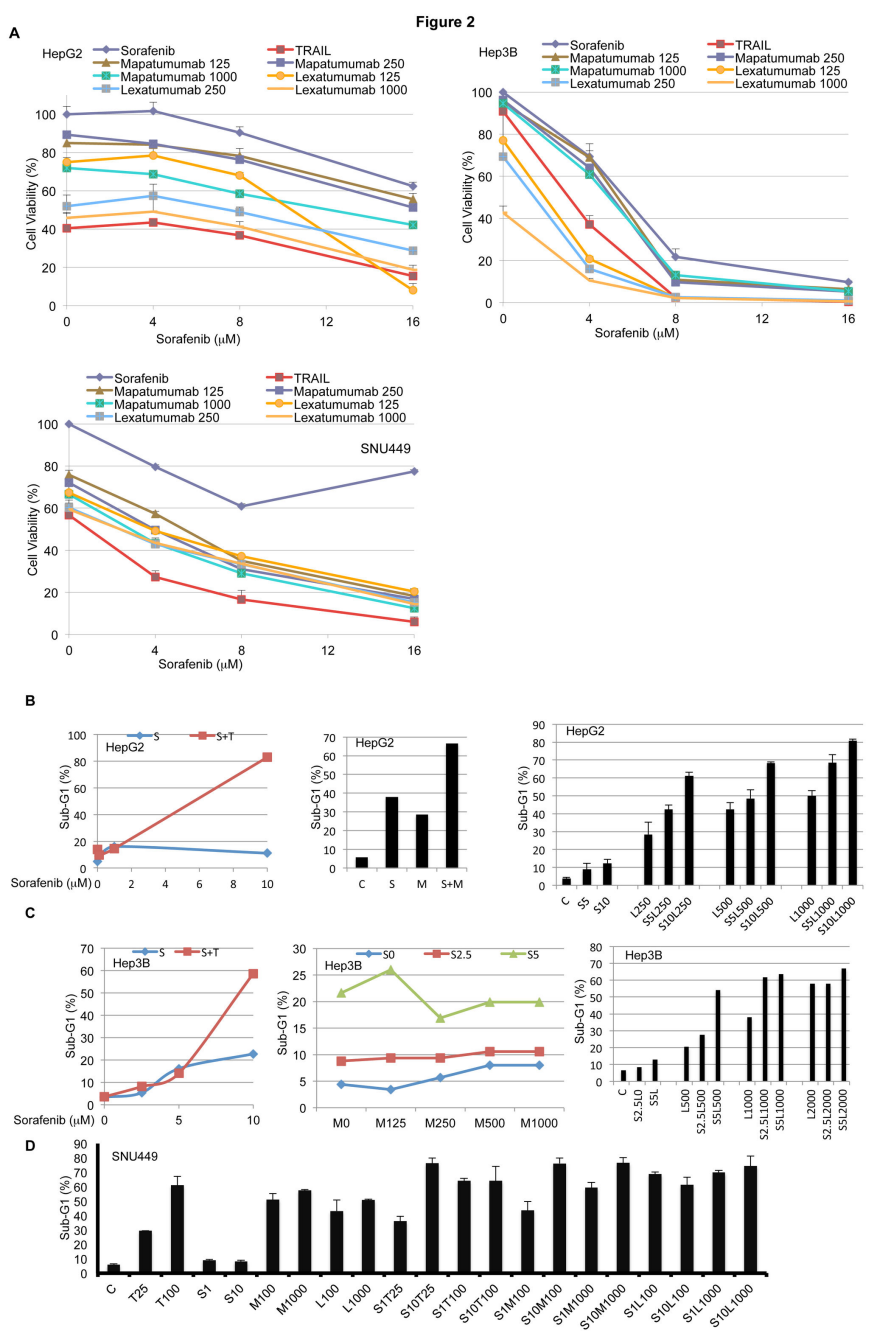
Sorafenib sensitizes human HCC cell lines to cell death induced by Apo2L/TRAIL and TRA antibodies. A. HepG2, Hep3B and SNU449 cells were treated with sorafenib (4, 8, 12, 16 µM), Apo2L/TRAIL (25 ng/mL), mapatumumab (125, 250 and 1000 ng/mL) or lexatumumab for (125, 250 and 1000 ng/mL) for 24 h and cell viability was determined by CellTiter-Glo assay (n=3, mean+S.D.). B-D. HepG2, Hep3B and SNU449 cells were treated with varying concentrations of sorafenib in combination with Apo2L/TRAIL, mapatumumab or lexatumumab for 24 h and sub-G1 content was analyzed (C=Control, S=Sorafenib, T =TRAIL, M=Mapatumumab, L=Lexatumumab)..

We tested the efficacy of combining sorafenib with Apo2L/TRAIL, mapatumumab, or lexatumumab. We first analyzed the effect of these combinations in cell viability assays in liver cancer cell lines. We observed that these combinations cooperatively led to a decrease in cell viability (Figure 2A). To assess cell death, we performed Sub-G1 analysis on treated cells. Combining sorafenib with Apo2L/TRAIL in the Apo2L/TRAIL-resistant HepG2 cells caused cell death in a synergistic manner. Sorafenib in combination with mapatumumab and lexatumumab yielded an additive response in these cells (Figure 2B). In the Apo2L/TRAIL-resistant Hep3B cell line, sorafenib synergized with Apo2L/TRAIL and lexatumumab, while it yielded an additive effect with mapatumumab (Figure 2C). We further observed that sorafenib synergizes with Apo2L/TRAIL, mapatumumab and as lexatumumab in SNU449 cells (Figure 2D). These hepatocellular carcinoma cell lines were treated with sorafenib, Apo2L/TRAIL, mapatumumab or lexatumumab for 72 h, washed with PBS and stained with crystal violet, and these experiments showed similar results (See Figure S1).

### Sorafenib in combination with Apo2L/TRAIL or Apo2L/TRAIL receptor agonist antibodies enhances cell death of solid tumor cell lines *in vitro*

**Figure 3:**
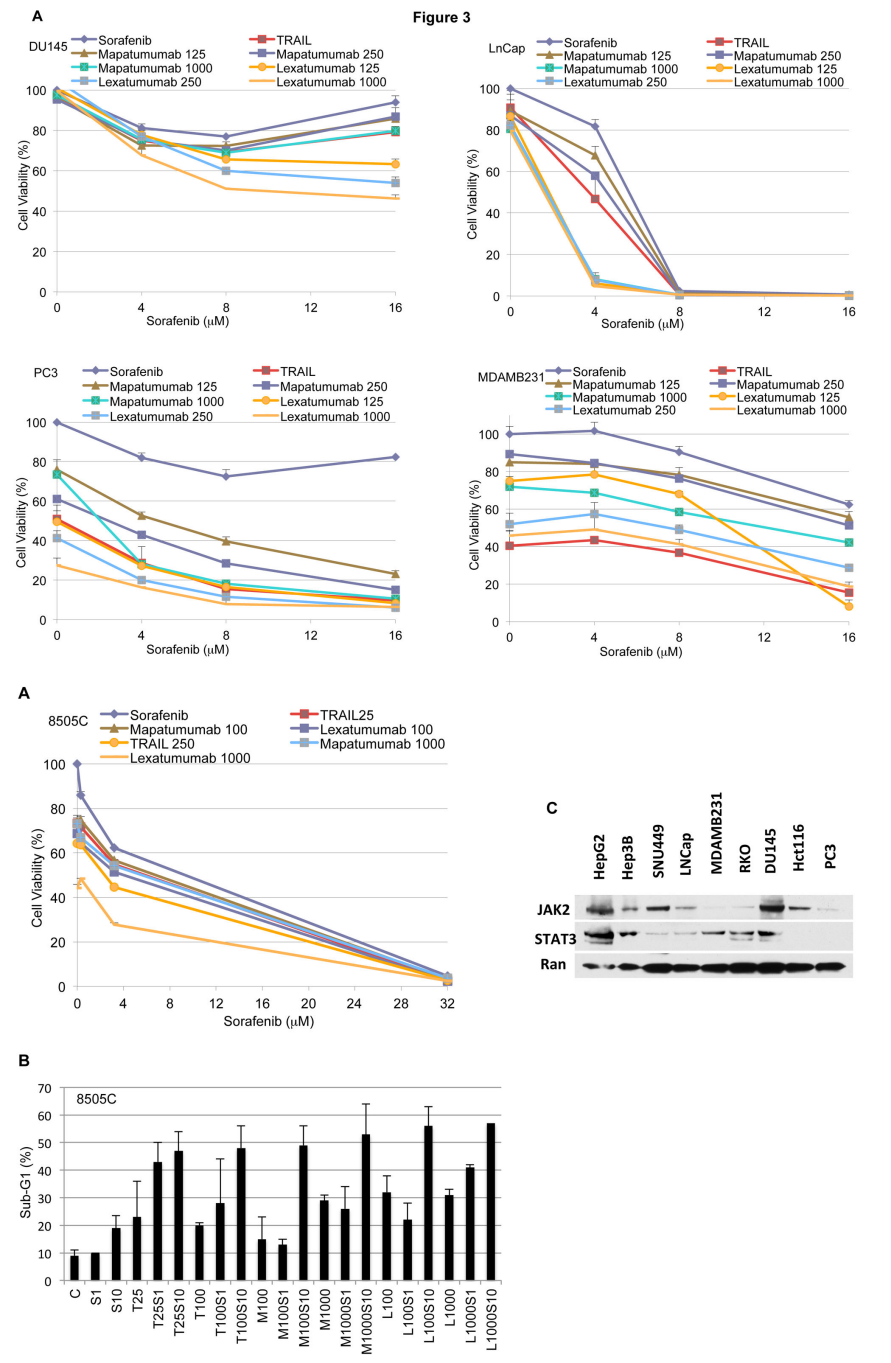
Sorafenib in combination with Apo2L/TRAIL and TRA antibodies enhances cell death of solid tumors *in vitro*. A. Panel of prostate, breast, colon and thyroid cancer cell lines was treated with different concentrations of these drugs in combination with sorafenib for 24 hr and growth analyzed by CellTiter-Glo (n=3, mean+S.D). B 8505C thyroid cancer cells were treated with varying concentrations of sorafenib (1, 10 µM), Apo2L/TRAIL (25, 100 ng/mL), mapatumumab (100, 1000 ng/mL) or lexatumumab (100, 1000 ng/mL). C. A panel of cell lines was harvested and lysates prepared for Western blot analysis for total Stat3 and Jak2 levels.

Once we determined that sorafenib sensitizes hepatocellular carcinoma cell lines to Apo2L/TRAIL *in vitro*, we wanted to evaluate the effect of combination of sorafenib with Apo2L/TRAIL, mapatumumab or lexatumumab in other solid tumor cell lines. Prostate carcinoma causes the second most cancer-related deaths in men in the United States [1]. We therefore evaluated a panel of prostate cancer cell lines, including PC-3, DU-145 and LNCap, for growth inhibition using different concentrations of sorafenib, Apo2L/TRAIL, mapatumumab or lexatumumab for 24 hours. We observed that the combination of sorafenib with Apo2L/TRAIL or Apo2L/TRAIL receptor agonist antibodies decreases cell viability (Figure 3A). Furthermore, all 3 prostate cancer cell lines treated with sorafenib, Apo2L/TRAIL, mapatumumab or lexatumumab for 72 hours, showed similar results by crystal violet staining (Figure S2).

Excluding non-melanoma skin cancer, breast cancer is the most common cancer among women in the United States [1] We therefore analyzed the human breast cancer cell line MDA-MB-231 *in vitro* (Figure 3A). We observed that the cell growth of the MDA-MB-231 cell line was inhibited with increasing concentrations of sorafenib, Apo2L/TRAIL, mapatumumab or lexatumumab as single agents as well as in combination (Figure 3A).

The overall 5-year survival rate of anaplastic thyroid carcinoma is 14% [21]. We therefore tested these agents in the human anaplastic thyroid carcinoma 8505C cell line. We observed that these combinations decrease the cell viability in 8505C cells (Figure 3A). We confirmed cell death by sub-G1 analysis in the 8505C cell line (Figure 3B). We observed synergy with sorafenib and mapatumumab; and an additive effect with sorafenib and Apo2L/TRAIL or lexatumumab (Figure 3B).

We analyzed the expression of JAK2/STAT3 in most of the cell lines (Figure 3C). However, there was no clear correlation with the sensitivity/resistance of these cell lines. We used the Chou Talalays method to determine synergy [22]. See tables 1 and 2 summarizing this synergistic effect.

**Table 1:** Sorafenib and Apo2L/TRAIL/TRA act in a synergistic manner in a panel of solid tumor cell lines: Calcusyn analysis of solid tumor cell lines that were treated with sorafenib and Apo2L/TRAIL/TRA in Figures 2 and 3 that were analyzed by CellTiter-GLO.

	**HepG2**	**Hep3B**	**SNU449**	**LNCap**	**231**	**DU145**	**PC3**
**S4T25**	1.149	0.365	0.207	0.822	1.433	0.385	0.324
**S8T25**	0.974	0.557	0.092	0.731	1.303	0.357	0.129
**S16T25**	0.972	0.179	0.026	1.188	0.991		
**S4M125**	0.826	0.826	0.201	0.951	0.822	0.266	0.521
**S4M250**	0.795	0.609	0.126	0.881	1.385	0.393	0.577
**S4M1000**	0.71	0.705	0.095	0.555	0.824		
**S8M125**	1.236	1.236	0.101	0.81	0.849	0.517	0.381
**S8M250**	1.065	1.065	0.079	0.691	0.951	0.389	0.345
**S8M1000**	1.182	0.399	0.07	0.691	0.754		
**S16M125**	0.73	0.73	0.06	1.188	1.118		
**S16M250**	0.711	0.529	0.052	1.188	1.093		
**S16M1000**	0.731	0.534	0.032	1.188	1.051		
**S4L125**	0.726	0.279	0.124	0.528	2.593	0.576	0.164
**S4L250**	0.733	0.257	0.083	0.549	1.019	0.523	0.128
**S4L1000**	1.235	0.258	0.098	0.503	1.898	0.217	0.253
**S8L125**	0.483	0.19	0.115	0.712	1.442	0.235	0.068
**S8L250**	0.464	0.198	0.092	0.731	0.92	0.135	0.047
**S8L1000**	0.497	0.19	0.093	0.731	1.393	0.095	0.052
**S16L125**	0.81	0.262	0.072	1.188	0.722		
**S16L250**	0.766	0.262	0.044	1.188	0.999		
**S16L1000**	0.266	0.198	0.04	1.27	0.957		

The resulting Combination Index (CI) of Chou-Talalay indicates additive effect (CI=1), synergism (CI<1), and antagonism (CI>) in drug combinations.

**Table 2:** Sorafenib and Apo2L/TRAIL/TRA act in a synergistic manner in 8505C thyroid cancer cell line: Calcusyn analysis of solid tumor cell lines that were treated with sorafenib and Apo2L/TRAIL/TRA in Figure 3 that were analyzed by CellTiter-GLO.

	**8505C**
**S0.3T25**	0.974
**S0.3T250**	1.094
**S3.2T25**	1.549
**S3.2T250**	1.046
**S32T25**	0.365
**S32T250**	0.38
**S0.3M100**	10.751
**S0.3M1000**	0.237
**S3.2M100**	1.651
**S3.2M1000**	1.508
**S32M100**	0.482
**S32M1000**	0.541
**S0.3L100**	0.87
**S0.3L1000**	1.569
**S3.2L100**	1.539
**S3.2L1000**	0.693
**S32L100**	0.322

The resulting Combination Index (CI) of Chou-Talalay indicates additive effect (CI=1), synergism (CI<1), and antagonism (CI>) in drug combinations.

There is an estimated 50,000 and 150,000 deaths due to colorectal and lung carcinomas respectively in the United States each year [1]. We analyzed these drugs in colon (HCT116 Bax^-/-^, HCT116) and lung (H460) cancer cell lines (Figure S3). Apo2L/TRAIL, mapatumumab or lexatumumab had single agent activity against the HCT116 as well as the H460 cells, while the HCT116 Bax^-/-^ cells were resistant, as expected. The HCT116 Bax^-/-^ cells were sensitized to cell death by combinations of sorafenib plus Apo2L/TRAIL, mapatumumab, or lexatumumab.

### Sorafenib inhibits the Jak2/Stat3/Mcl-1 axis

**Figure 4:**
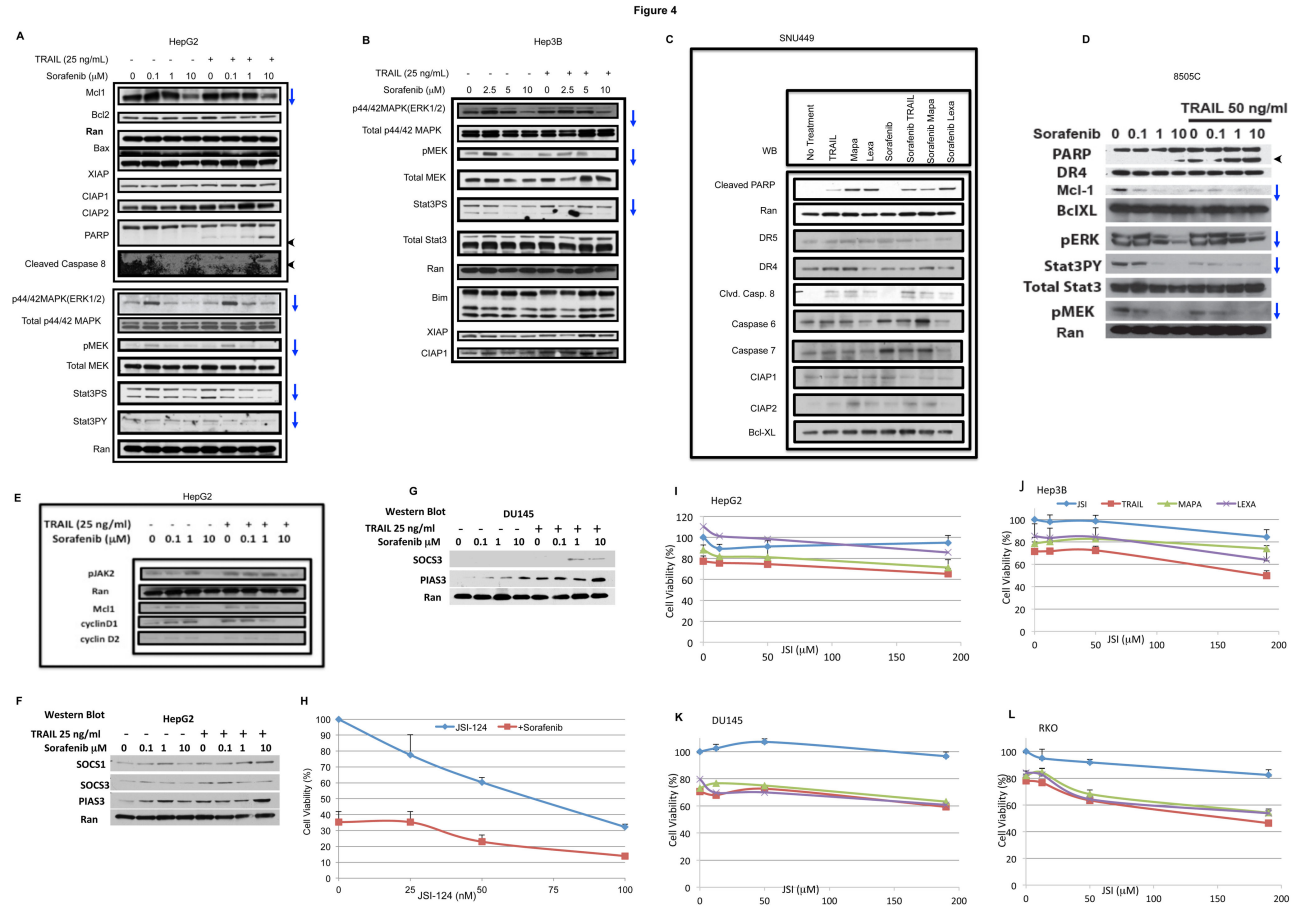
Sorafenib mediates apoptosis by inhibiting Stat3 pathway. A. HepG2 cells were treated with sorafenib (0.1, 1, 10 µM) and Apo2L/TRAIL (25 ng/mL) cells. Western blot (WB) analysis was done to observe for cell death (PARP cleavage and caspase 8), pERK, pMEK, Stat3PY and Stat3PS. B. Hep3B cells were treated with sorafenib (0.1, 1, 10 µM) and Apo2L/TRAIL (25 ng/mL) cells. WB analysis was performed to analyze the expression of pERK, pMEK and Stat3PS. C. SNU449 cells were treated with sorafenib (10 µM), Apo2L/TRAIL (25 ng/mL), mapatumumab (100 ng/mL) or lexatumumab (100 ng/mL) for 2 h and cell lysates prepared for WB analysis. D. 8505C cells were treated with sorafenib (0.1, 1, 10 µM) and Apo2L/TRAIL (50 ng/mL) and cell lysates prepared for WB analysis. E. HepG2 cells were treated with sorafenib (0.1, 1, 10 µM) and Apo2L/TRAIL (25 ng/mL) for 4 hrs. WB analysis was done to screen for proteins upstream regulators (pJAK2) and downstream modulators of Stat3 namely cyclin D1, cyclin D2 and Mcl-1. F and G. HepG2 and DU145 cells were treated with with sorafenib (0.1, 1, 10 µM) and Apo2L/TRAIL (50 ng/mL) and cell lysates prepared for WB analysis of SOCS and PIAS. Figure 4H. Hep3B cells were treated with JSI-124 (Cucurbitacin I) (25, 50, 100 nM) in combination with sorafenib (3.2 µM) for 24 h and cell viability was assessed by CellTiterGlo. I-L. A panel of cell lines were treated with JSI-124 (12.5, 50 and 190 nM) in combination with Apo2L/TRAIL (25 ng/ml) or lexatumumab/mapatumumab (200 ng/ml) for 14 h and cell viability assessed by CellTiterGlo.

Once we found that the combination of sorafenib with Apo2L/TRAIL, mapatumumab or lexatumumab cooperatively causes cell death *in vitro*, we further investigated the underlying mechanism. We corroborated induction of apoptosis by assaying the cells for PARP cleavage and cleaved caspase-8 by Western blot analysis in HepG2, SNU449 and 8505C cell lines (Figure 4A, 4C and Figure 4D). The cleaved products were increased in a concentration-dependent manner. Since sorafenib is a multikinase inhibitor, we analyzed phospho-ERK and phospho-MEK and found those to be downregulated in a concentration-dependent manner (Figure 4A, 4B and 4D). Furthermore the active forms of Stat3 (Stat3PY^705^ and Stat3PS^727^) are downregulated in a concentration-dependent manner in cells treated with sorafenib (Figure 4A, Figures 4B and 4D). However, the total levels of Stat3, MEK and ERK were not downregulated. Because the active forms of Stat3 were downregulated, we analyzed the pathway upstream and downstream of Stat3.

Stat3 is phosphorylated at residue Tyr^705^ as well as Ser^727^. This phosphorylation is mediated by receptor-associated tyrosine kinases, such as JAKs [23]. We therefore analyzed the expression of the active form of Jak2 (pJAK2Tyr^1007/1008^) and found it to be downregulated. Stat3 may promote cell proliferation by upregulating cyclin D1 and c-myc; and may suppress apoptosis by downregulating survivin and Bcl-XL [24,25,26]. We further characterized the downstream pathway of Stat3 and determined that Mcl-1, cyclin D1, and cyclin D2 were downregulated in HepG2 cell lines in a concentration-dependent manner (Figure 4E). In the cell lines tested, sorafenib did not downregulate the anti-apoptotic proteins Bcl-2 or Bcl-XL. Also, there was no change in caspase inhibitor protein family members: c-IAP-1, c-IAP-2, or XIAP. The levels of death receptors, DR4 and DR5, were also not affected in the cell lines tested. In agreement with the inhibitory effect of sorafenib on the JAK/STAT pathway, we also observed that the negative regulators of JAK-STAT pathway SOCS and PIAS are upregulated when treated with sorafenib and TRAIL (Figure 4F and G).

We then investigated the effect of combination of JSI-124 (Cucurbitacin I), a selective inhibitor of Jak-Stat3, in combination with sorafenib for 24 hours. We observed that it decreased the cell viability in Hep3B cell lines (Figure 4H). We further observe that combining JSI-124 with Apo2L/TRAIL/TRA cooperatively decreased cell viability in a panel of solid tumors (Figure 4I-L). Our findings suggest that the Jak2-Stat3-Mcl1 axis maybe a common mechanism to be downregulated by sorafenib in a variety of human solid tumors of different tissue origins.

### Apo2L/TRAIL and Apo2L/TRAIL receptor agonist antibodies inhibit tumor growth *in vivo*

In addition to *in vitro* characterization of cell death and mechanism, we also confirmed these findings *in vivo*. For the *in vivo* studies we analyzed one prostate (DU-145), liver (HepG2), breast (MDA-MB-231) and colon (RKO) cancer cell line. Mice bearing tumor xenograft transplants were treated with sorafenib at 30 mg/kg daily for 5 days, Apo2L/TRAIL 100 µg i.v. every two days for 3 doses, or Apo2L/TRAIL receptor-agonist antibodies at 10 mg/kg every two days for 3 doses.

**Figure 5:**
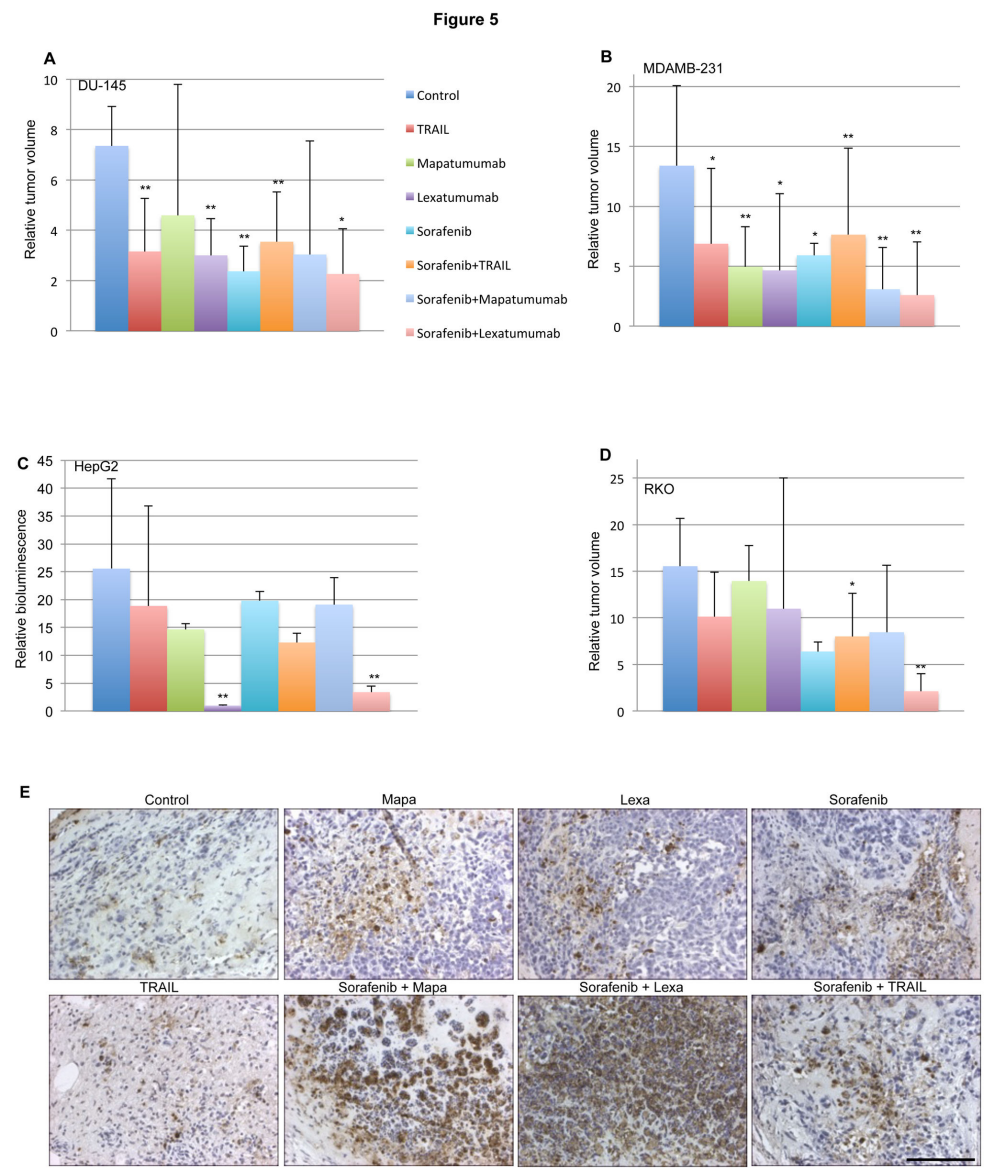
Sorafenib, Apo2L/TRAIL, mapatumumab and lexatumumab are effective in delaying tumor growth *in vivo*. A-D. Effects of combinatorial therapy on DU145 (A), MDA-MB-231 (B), HepG2(C) and RKO (D) xenografts in nu/nu mice (n=3-7 mice/group). In A, B and D tumor growth was monitored by caliper measurements while in C by bioluminescence imaging (C). Statistical significance was performed using an unpaired, two-tailed Student’s t-test comparing the control with the treated mice at the end point (*p<0.05, **p<0.005). Mice were monitored for signs of overt toxicity and weight loss. E. Tissue from mice was harvested at 48 h after treatment and TUNEL assay performed.

We observed that a combination of lexatumumab and sorafenib delayed tumor growth in all of the solid tumor xenografts: prostate, DU145 (Figure 5A); breast, MDA-MB-231 (Figure 5B); liver, HepG2 (Figure 5C); and colon cancer, RKO (Figure 5D). In addition, in DU145 xenografts we observed that Apo2L/TRAIL, lexatumumab, sorafenib and sorafenib +Apo2L/TRAIL delayed tumor growth (Figure 5A). We found delayed tumor growth in MDA-MB-231 xenografts with all agents either as monotherapies or in combination (Figure 5B).

Furthermore, HepG2 xenografts were inhibited by lexatumumab as a single agent (Figure 5C). After 12 days of initiating treatment, there was a complete regression among lexatumumab treated tumors. There was a decrease in the tumor size on treatment with sorafenib and mapatumumab, but it was not significant at day 12.

In RKO xenografts, we found that sorafenib plus Apo2L/TRAIL therapy delayed tumor growth (Figure 5D). Mice were monitored for overt toxicity and body weight loss. Mice in the RKO xenograft experiment did not exhibit any significant weight loss (Figure S4). No weight loss or gross toxicity was observed in any other xenograft studies (data not shown).

Tumors were harvested 48 hours after initial treatment and analyzed by immunohistochemical staining. We observed an increased amount of TUNEL staining in the sorafenib plus lexatumumab therapy combination in the RKO xenograft tumors in the outer border of the tumor margins as compared to the untreated control xenograft tumors (Figure 5E).

## Discussion

Solid tumors cause significant morbidity and mortality mostly due to metastasis, lack of response to therapy or because of an un-resectable tumor mass. There are concerted efforts to improve chemotherapeutic efficacy by rationally designing drugs that would specifically inhibit essential molecular targets within the cancer cell. It is therefore crucial to selectively target cancer cells while having no effect on normal cells. Apo2L/TRAIL is one of the pathways that leads to tumor cell death and tumor suppression *in vivo*, but about half of tumor cell lines are Apo2L/TRAIL-resistant. While soluble recombinant Apo2L/TRAIL may bind to the decoy receptors as well, antibodies targeting specific death receptors bind to their specific apoptosis-inducing receptor [3]. Apo2L/TRAIL or Apo2L/TRAIL-Receptor agonist antibodies can be combined with other drugs and are currently undergoing phase I and phase II clinical trials [6,7,8].

Sorafenib (Nexavar), a multikinase inhibitor, was originally developed as a RAF inhibitor but has subsequently been shown to inhibit a number of other kinases. Sorafenib was approved by the FDA for the treatment of advanced renal carcinoma in 2005 and unresectable liver carcinoma in 2007 [10,11]. There are currently over 200 open clinical trials of sorafenib in combination with other therapies (www.clinicaltrials.gov). We are the first group to report (a) the effect of combination of sorafenib and Apo2L/TRAIL, or the DR4 and DR5 agonist antibodies (mapatumumab and lexatumumab, respectively) in a panel of solid tumor cell lines both *in vitro* and *in vivo* (b) to suggest that Jak2-Stat3-Mcl1 axis maybe a common mechanism to be down-regulated by sorafenib in a variety of human solid tumors of different tissue origins.

We observed that sorafenib sensitizes Apo2L/TRAIL-resistant cell lines to cell death both *in vitro* and *vivo*. Activation of DR4 and DR5 with TRA in combination with sorafenib elicited a different profile of apoptotic response. In tumor cell lines at several concentrations and time points, we found that lexatumumab is a potent inducer of cell death. Apo2L/TRAIL-resistant HepG2 cells treated with TRA agonist antibody lexatumumab at 10 mg/kg body induced a complete disappearance of tumors within 12 days (Figure 5C). When we treated HepG2 cells *in vitro* we found that treatment with lexatumumab (125 ng/mL) in combination with sorafenib (8 µM) decreases cell viability in most of the cells at the 24 hour time point and the combination of mapatumumab (125 ng/mL) required sorafenib (16 µM) to obtain similar results (Figure 3A). Furthermore, lexatumumab caused an inhibition of tumor growth in combination with sorafenib in known Apo2L/TRAIL-resistant cell lines (RKO) *in vivo*. The MDA-MB-231 cell line is considered to be both Apo2L/TRAIL-resistant and Apo2L/TRAIL sensitive [27,28]. We found it to be sensitive to Apo2L/TRAIL, sorafenib, mapatumumab or lexatumumab as single agents, both *in vitro* and *in vivo*.

In order to determine if the level of DR4 and DR5 surface receptors has any effect on the sensitivity of these cell lines we analyzed a panel of solid cancer cell lines (Figure S5). SNU449 is sensitive to Apo2L/TRAIL as well as mapatumumab or lexatumumab and expresses high levels of their target surface death receptors. RKO is relatively resistant to Apo2L/TRAIL, mapatumumab and lexatumumab as a single agent and also expresses significantly higher concentration of DR4 and DR5. Therefore, we posit that the surface expression of DR4 and DR5 receptors may not fully explain Apo2L/TRAIL resistance. DR5 contributes more than DR4 to TRAIL-induced apoptosis in cells that express both the death receptors [29]. However, this effect may be cell specific, as CLL cells demonstrate a preferential signaling for DR4 over DR5 [30]. We think there might be a preferential signaling for DR5 in some of these cell lines that we tested, which may indicate an enhanced cell kill effect for lexatumumab over mapatumumab at the concentrations that we analyzed. Others indicate that lexatumumab might be more effective than mapatumumab [31,32]. There are newer TRAIL based therapies in the pipeline. Our work here would be extended to emerging TRAIL-based agents such as small molecules that boost production of TRAIL or induce DR5 clustering as well as protein scaffolds designed to engage the TRAIL receptors [33,34,35,36].

We observed that inhibition of cell growth by sorafenib downregulates the active forms of Stat3, Stat3Tyr^705^ and Stat3Ser^727^. Stat3 is an oncogene and is constitutively active in a number of solid and hematological malignancies. Furthermore, Stat3 also plays a role in metastasis [37]. Stat3 mediated transcription plays a role in cell survival and cell-cycle progression. IL-6 binds to the IL-6 receptor (IL-6), this complex then associates with gp130. This binding causes receptor dimerization, activating Jaks that phosphorylate themselves and the receptor. We observed that sorafenib downregulates pJAK2 expression. The receptor site on Jak serves as a docking site for Stat3. Receptor-bound Stats phosphorylated by Jaks dimerize and translocate in to the nucleus to regulate gene transcription. We observed that the active forms of Stat3, Stat3Tyr^705^ and Stat3Ser^727^ are decreased while the total Stat3 protein levels are not affected. Stat3 plays a role in the cell cycle and key genes in the cell cycle, including cyclin D1 and cyclin D2. The expression of cyclin D1 and cyclin D2 protein level is decreased by sorafenib (Figure 4E). Stat3 also plays a role in cell survival and expression of Mcl-1, a member of the bcl-2 family, a known apoptotic protein. We have previously shown that sorafenib downregulates Mcl-1 levels in colon cancer. In addition to the multikinase inhibitory effect of sorafenib on the JAK/STAT pathway, we also observe that the negative regulators of JAK-STAT pathway SOCS and PIAS are upregulated when treated with sorafenib and TRAIL (Figure 4F and G). SOCS can inhibit JAK/STAT signaling pathways in three ways. First, SOCS can bind the receptor phosphotyrosines and physically block recruitment of STATs. Second, SOCS binding to JAKs/receptors can inhibit the JAK kinase activity. Third, SOCS may facilitate ubiquitination of JAKs and their receptors leading to proteosomal degradation. PIAS proteins bind to activated STAT dimers, thereby inhibiting STAT binding to the DNA.

Furthermore, when we combine sorafenib (3.2 µM) with JSI-124 (Cucurbitacin I) (25, 50, 100 ng/mL), a selective known Jak2-Stat3 inhibitor, it decreases cell viability (Figure 4H). We also observe that the combination of JSI-124 with Apo2L/TRAIL/TRA decreases cell viability. These findings suggest Stat3 is a molecule downregulated by sorafenib, and its downregulation may potentially lead to enhanced cell death. Stat3 is a target for therapy. A phase I study of Stat3 inhibitor in solid tumors at M D Anderson Cancer Center (NCT00955812) and a phase 0 study of a Stat3 decoy in head and neck cancer was recently completed (NCT00696176) (www.clinicaltrials.gov)

Based on our findings we suggest the following: (a) Sorafenib may have a role in combination with other standard therapies in colon, breast, prostate and thyroid cancer, (b) Mapatumumab or lexatumumab maybe combined with sorafenib in therapy of solid cancers. (c) Stat3 is a candidate for targeted therapy in combination with current drug regimens in solid tumors and Stat3 inhibition may be worth further investigation in combinatorial therapies targeting the Apo2L/TRAIL pathway.

## Materials and Methods

### Reagents and antibodies

Sorafenib (Nexavar) was synthesized at the Medical University of Southern Carolina by Dr. Charles D. Smith. Apo2L/TRAIL receptor agonist antibodies DR4 (mapatumumab) and lexatumumab (DR5) were provided by Dr. Robin Humphreys. His Tag-recombinant human ApoL/TRAIL was produced and purified as described earlier [38]. For *in vitro* experiments sorafenib was dissolved in DMSO whereas for *in vivo* studies it was dissolved in cremophor/ethanol/water solution (Cremophor EL 12.5%, ethanol 12.5%, water, 75%) as previously described [39]. Stat3 siRNA was obtained from Cell Signaling Technology, Beverly, MA. JSI-124 (Cucurbitacin I), Jak/Stat3 inhibitor was obtained from the National Cancer Institute, Bethesda, MD.

The following antibodies were used: Caspase 8, PARP, cyclin D1, cyclin D2, Stat3-PY, Stat3-PY, Stat3, pERK, pMEK, ERK, MEK, Mcl-1, Jak-2, pJAK2, SOCS, PIAS (Cell Signaling Technology, Beverly, MA); Ran, XIAP (BD Pharmingen, San Diego, CA). All antibodies were diluted to 1:1000, except Ran (1:5000)

### Cell Culture

The following cell lines (HepG2, Hep3B, SNU449, DU145, PC-3, LNCap, MDA-MB-231, RKO, HT29, Hct116) were purchased from American Type Culture Collection while 8505C cells [40] was generously provided by Dr. Patricia Mclaughlin at Penn State Hershey Cancer Institute. All these cell lines were propagated in a stable humidified incubator maintained at 37°C and 5% CO2 in the recommended media-supplemented with 10% fetal bovine serum and 100 µg of penicillin/streptomycin (CORNING Cellgro, Manassas, VA). We regularly use antibiotics in our cell culture media and follow all the standard aseptic precautions as is recommended in a BSL2 lab. We test the cells for mycoplasma only when we expect some source of contamination.

### Cell viability and death analysis

Cells were plated in a clear bottom, black wall 96-well plate in 100 µL of media for the cells to attach. After overnight incubation the cells were treated with different concentrations of sorafenib, Apo2L/TRAIL, mapatumumab or lexatumumab for varying time points (14, 24, 48 and 72 h) as needed. Cell viability was assessed by CellTiter-Glo assay (Promega) according to the manufacturer’s protocol. Bioluminescence was recorded on the IVIS imaging system. Sub-G1 content was determined by propidium iodide (PI) staining for DNA content and FACS. Floating and adherent cells were collected and fixed in ethanol, followed by RNAse treatment and PI staining. Flow Cytometry samples were run in Beckman Coulter and analyzed using the Epic Elite Flow Cytometry Workstation ver.4.5, Hialeah, FL.

### Western blot analysis

For Western blot analysis, cells were physically disrupted and lysed in the presence of protease and phosphatase inhibitors. The protein concentration was measured by the Bio-Rad Protein Assay (Bio-Rad laboratories, Hercules, CA), according to the manufacturer’s instructions. Equal amounts of proteins were loaded and electrophoresed on a 4-12% SDS-polyacrylamide gel using the XCell system (Invitrogen). Proteins were transferred to an Immobilon-P PVDF membrane (Millipores) with a wet transfer apparatus (Bio-Rad) for 2h @ 200mA. Membranes were blocked with 10% (w/v) non-fat dry milk in TBST, incubated with the primary antibody overnight at 4°C, and subsequently with horse-radish peroxidase-labeled secondary antibody for 2 h at room temperature. The signal was visualized by Chemiluminescent substrate and X-ray film.

### Tumor xenograft experiments

Four to six week old female athymic NCr-nu/nu mice (Charles River, Germantown, MD) were used for animal studies. The mice were housed and maintained in accordance with the Pennsylvania State University College of Medicine Institutional Animal Care and Use Committee and state and federal guidelines for the humane treatment and care of laboratory animals. This study was approved by the IACUC Committee (2010-32). For each xenograft study, 2.5 million cell were mixed with Matrigel (BD Biosciences, Bedord, MA) making a 200 µL suspension (50% cell suspension in PBS, 50% Matrigel) and injected subcutaneously to the right and left rear flanks of the mice. Cells were allowed to grow when the volume reached ~100mm^3^. Tumor volumes were determined using the following formula [Volume = 0.52x (width)^2^ x length]. At that point mice were treated with sorafenib (12.5% Cremophor EL/12.5% ethanol/ 75% water) (30 mg/kg body weight daily IP) (daily x 5 doses), Apo2L/TRAIL (100 μg/mouse IV) (alternate day-3 doses) and Apo2L/TRAIL agonist antibodies (10 mg/kg body weight) (alternate day-3 doses). Tumor progression was monitored and quantified using 2 different methods. The tumor size was measured by digital caliper measurements and relative tumor size (compared to the original tumor size at the time of the treatment) was used to determine the dose response. For HepG2 cells infected with luciferase, noninvasive bioluminescence (photons/sec/cm^2^) signals were visualized by intraperitoneal injection of 5 mg D-luciferin into anesthetized mice (ketamine/xylazine), followed by detection of images using a Xenogen IVIS system [41].

### Histology and Immunohistochemistry

Excised tumors were harvested 48 hours after treatment and fixed in 4% paraformaldehyde. Fixed tumors were paraffin embedded and sectioned. TUNEL assays were performed according to the manufacturer’s protocol with the ApopTag Perioxidase In Situ Apoptosis detection Kit (Millipore) and DAB peroxidase substrate kit (Vector Laboratories).

### Surface death receptors, DR4 and DR5 expression

For DR4 and DR5 surface expression experiments, cells were grown in log phase in 6-well plates under ATCC recommended conditions. Cells were harvested by brief trypsinization, washed with PBS, and fixed for 30 minutes with 4% paraformaldehyde in PBS. Cells were washed twice with PBS and incubated with the primary antibody or an equivalent amount of isotype antibody for 1 hour at room temperature. The primary antibodies, αDR5 (IMG-120A, Imgenex) and αDR4 (IMG-141, Imgenex) were used at a dilution of 1:200 in PBS. Cells were incubated with anti-rabbit IgG and anti-mouse IgG1 Alexa Fluor secondary antibodies at 1:250 in PBS for 30 minutes at room temperature. Cells were washed twice in PBS and resuspended in PBS for immediate analysis by flow cytometry.

### Statistical Analysis

Statistical analysis was performed using an unpaired, two-tailed Student’s t-test. All comparisons were made relative to untreated controls, and statistically significant differences are indicated as *p<0.05 and **p<0.005. We used Calcusyn software (which utilizes the Chou Talalay’s method) to determine synergy.

## Supporting Information

Figure S1 **Sorafenib in combination with Apo2L/TRAIL and TRA antibodies enhances cell death in a panel of HCC cell lines.** A panel of liver cancer cell lines was treated with different concentrations of these drugs in combination with sorafenib. Cells were washed after 48 h and stained with crystal violet.
(TIFF)

Figure S2 **Sorafenib in combination with Apo2L/TRAIL and TRA antibodies enhances cell death in a panel of prostate cancer cell lines.** A panel of prostate cancer cell lines was treated with different concentrations of these drugs in combination with sorafenib. Cells were washed after 48 h and stained with crystal violet.
(TIFF)

Figure S3 **Sorafenib in combination with Apo2L/TRAIL and TRA antibodies enhances cell death in a colon and lung carcinoma cell lines.** Colon (Hct-116 and Hctbax^-^/^-^) and lung (H460) cancer cell lines were treated with different concentrations of these drugs in combination with sorafenib. Sub-G1 analysis calculated at 24 h.
(TIFF)

Figure S4 **Sorafenib in combination with Apo2L/TRAIL and TRA antibodies does not cause toxicity in mice.** Mice with RKO xenografts were weighed to observe for weight loss.
(TIFF)

Figure S5 **Analysis of surface death receptors, DR4 and DR5 in a panel of solid tumor cell lines.** Alexa fluor antibodies were targeted at the surface death receptors, DR4 and DR5; and analyzed by flow cytometry.
(TIFF)
