# Supplementary figures and images for: Sorafenib Sensitizes Solid Tumors to Apo2L/TRAIL and Apo2L/TRAIL Receptor Agonist Antibodies by the Jak2-Stat3-Mcl1 Axis
Source: PLoS One. 2013 Sep 26;8(9):e75414. doi: 10.1371/journal.pone.0075414 (PMC3784419; doi:10.1371/journal.pone.0075414)

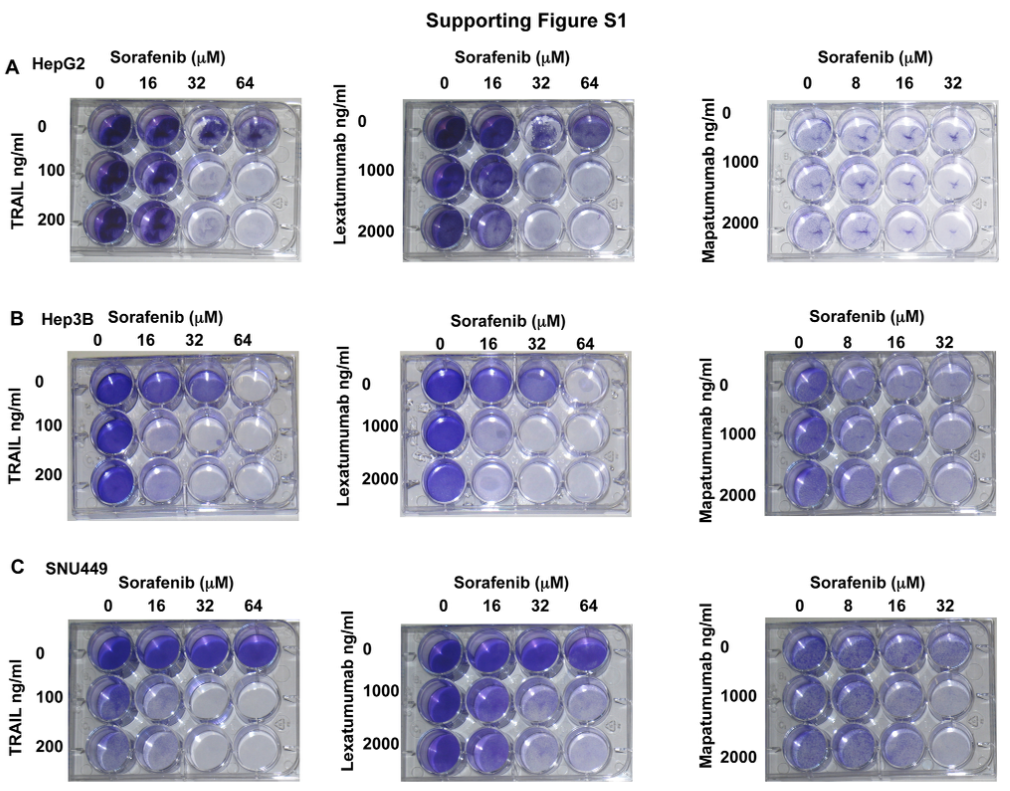

Supplement: Figure S1 — Sorafenib in combination with Apo2L/TRAIL and TRA antibodies enhances cell death in a panel of HCC cell lines. A panel of liver cancer cell lines was treated with different concentrations of these drugs in combination with sorafenib. Cells were washed after 48 h and stained with crystal violet. (TIFF) [file pone.0075414.s001.tiff]

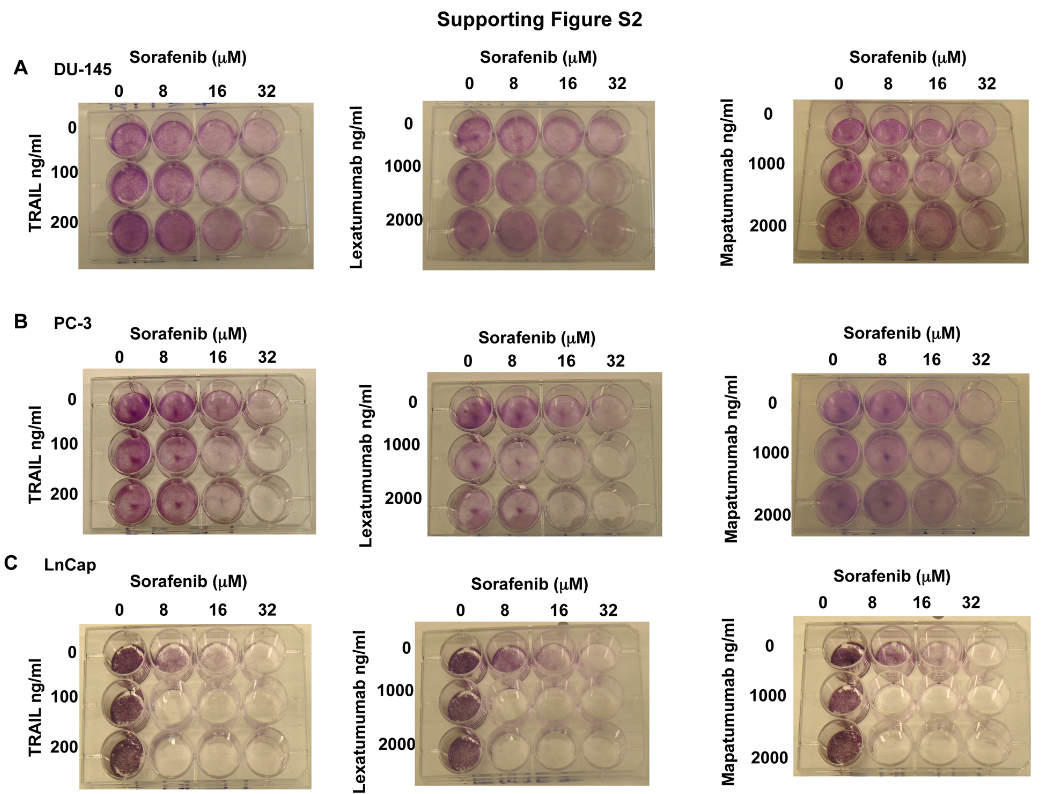

Supplement: Figure S2 — Sorafenib in combination with Apo2L/TRAIL and TRA antibodies enhances cell death in a panel of prostate cancer cell lines. A panel of prostate cancer cell lines was treated with different concentrations of these drugs in combination with sorafenib. Cells were washed after 48 h and stained with crystal violet. (TIFF) [file pone.0075414.s002.tiff]

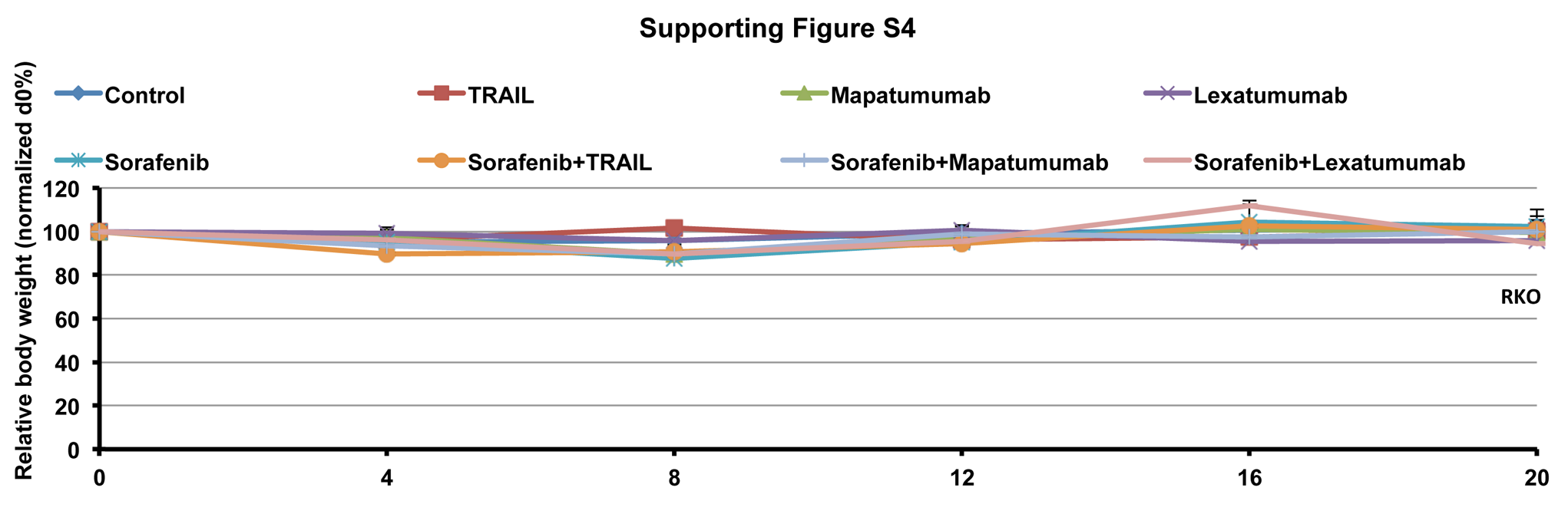

Supplement: Figure S4 — Sorafenib in combination with Apo2L/TRAIL and TRA antibodies does not cause toxicity in mice. Mice with RKO xenografts were weighed to observe for weight loss. (TIFF) [file pone.0075414.s004.tiff]

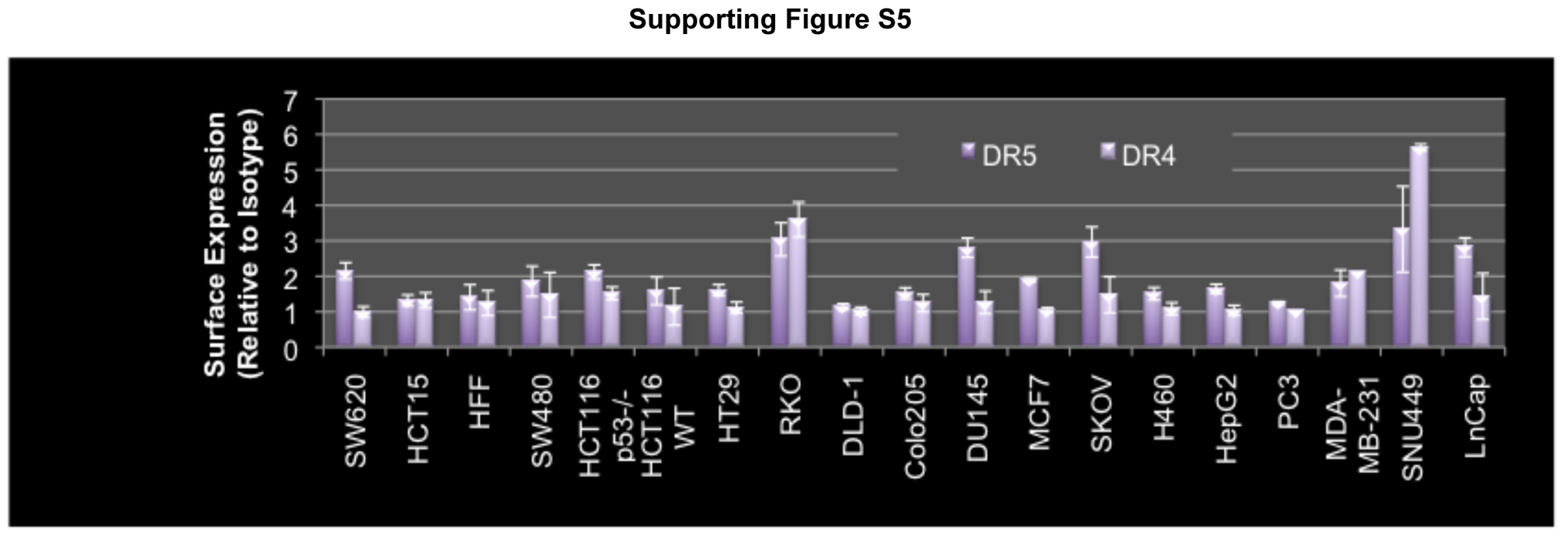

Supplement: Figure S5 — Analysis of surface death receptors, DR4 and DR5 in a panel of solid tumor cell lines. Alexa fluor antibodies were targeted at the surface death receptors, DR4 and DR5; and analyzed by flow cytometry. (TIFF) [file pone.0075414.s005.tiff]
